# Study design, rationale, and baseline characteristics of ATTAIN-HYPERTENSION, a phase 3 trial of the oral GLP-1 receptor agonist orforglipron for hypertension

**DOI:** 10.1016/j.ajpc.2026.101783

**Published:** 2026-08-06

**Authors:** Luke Laffin, Markus P. Schlaich, Michael E. Hall, Cathy C. Xie, Girish Rao, Mallik Rettiganti, Ellen B. Dennehy, Bruno Linetzky

**Affiliations:** a Cleveland Clinic Foundation, Cleveland, USA; b Dobney Hypertension Centre, Medical School, University of Western Australia, Royal Perth Hospital and RPH Research Foundation, Perth, Australia; c The University of Mississippi Medical Center, Jackson, USA; d Eli Lilly and Company, Indianapolis, USA

**Keywords:** Hypertension, Blood pressure, Orforglipron, GLP-1 RA, Pharmacotherapy, Randomized trial

## Abstract

**Background:**

Up to three-quarters of primary hypertension is epidemiologically attributed to excess adiposity. Incretin-based therapies are consistently associated with reduced blood pressure (BP) in clinical trials; however, no prior trial has investigated incretin-based therapies for hypertension treatment with BP as its primary efficacy outcome.

**Methods:**

ATTAIN—HYPERTENSION is a phase 3 master protocol directing two identical, randomized, placebo-controlled studies. Participants with BMI≥25kg/m^2^ and systolic BP (SBP)≥140 mmHg or diastolic BP (DBP)≥90 mmHg, independent of antihypertensive treatment, are randomized 2:1 to orforglipron or placebo. The study includes 5 periods: screening, a 4-week run-in during which treated participants change to standard background medications (olmesartan, amlodipine, hydrochlorothiazide), a 48-week treatment period including dose escalation and return to baseline antihypertensive medication, a 12-week randomized withdrawal period, and a 2-week safety follow-up. Primary end point is the change in office SBP at Week 36. Key secondary end points include change in ambulatory SBP, percent change in hsCRP, percent change in body weight, percent change in triglycerides and non-HDL cholesterol, and change in SBP across the randomized withdrawal period

**Results:**

ATTAIN—HYPERTENSION randomized 538 participants in Trial 1 and 546 in Trial 2 between April 2025 and January 2026 at 100 locations across 10 countries. The randomized population has a mean (SD) age of 53.7 (11.8) years, BMI of 35.6kg/m² (6.9), and office SBP of 144.2 mmHg (10.3). At screening, 55.4% of participants were treated with antihypertensive medication.

**Conclusion:**

ATTAIN—HYPERTENSION investigates the efficacy and safety of orforglipron for the treatment of hypertension in adults with overweight or obesity. Described here are the novel trial design, its rationale, and the enrolled population’s baseline characteristics. (ClinicalTrials.gov: NCT06948422)

## 1 Introduction

Hypertension is the leading cause of mortality globally, with 10.8 million deaths directly attributable to uncontrolled blood pressure (BP), representing >19% of total deaths [1]. Despite the availability of multiple well-established antihypertensive treatments, BP control rates remain poor and are decreasing. These trends are being driven by decreasing awareness and treatment rates [2], therapeutic inertia, suboptimal treatment intensification in clinical practice [3] and deprioritization of relevant pathophysiologic mechanisms.

Overweight or obesity affect over 2.5 billion people worldwide, increasing the risk of multiple health complications [4]. Epidemiological evidence indicates that 65% to 75% of primary hypertension cases are attributable to overweight or obesity [5]. Several mechanisms contribute to BP elevations among people with overweight and obesity, including kidney dysfunction associated with increased tubular sodium reabsorption through the accumulation of visceral, perirenal and renal sinus fat, activation of the renin–angiotensin–aldosterone system, increased renal sympathetic nerve activity (RSNA), impaired pressure natriuresis, among others [6,7]. Current approaches to achieve adequate BP control among people with hypertension often require a combination of several antihypertensive medications acting predominantly on the downstream effects of mechanisms influenced by adiposity and obesity, instead of the underlying obesity related pathophysiology [8]. Novel approaches targeting the underlying cause for sustained BP elevations in people with overweight or obesity represent an unmet clinical need.

Clinical guidelines from the United States, United Kingdom, and Europe recommend weight loss of at least 5% of body weight to prevent or treat hypertension in adults with overweight or obesity [[8], [9], [10], [11]]. The evidence for treatment of hypertension with weight management therapies from dedicated randomized clinical trials is limited. The ENCORE study demonstrated that the addition of exercise and weight loss to the Dietary Approaches to Stop Hypertension diet produced greater BP reductions compared with dietary modification alone in participants with overweight and obesity with above-normal BP [12]. The TONE trial showed that reduced sodium intake and weight loss constitute a feasible and effective nonpharmacologic strategy to maintain BP control following antihypertensive medication withdrawal in older adults [13]. The GATEWAY study proved that Roux-en-Y gastric bypass surgery combined with medical therapy was more effective than medical therapy alone in reducing the number of antihypertensive medications while maintaining controlled BP in people with obesity and hypertension [14].

Glucagon-like peptide-1 (GLP-1) receptor agonists (RA) and glucose-dependent insulinotropic polypeptide (GIP)/GLP-1 RAs have emerged as effective treatments for weight management in people with obesity and in people with overweight and other health-related conditions [14]. Accumulating evidence suggests that these medications may also reduce BP in participants with overweight or obesity, such that the 2025 US guidelines note that incretin mimetics, when used for weight management, may be effective as an adjunct to lower BP [15]. For example, in post hoc analyses of the SURMOUNT-1 trial and SURPASS clinical program, treatment with the GIP/GLP-1 RA tirzepatide was associated with reduced BP, and the underlying mechanisms appear to be both weight-dependent and weight-independent [[16], [17], [18]]. Furthermore, tirzepatide has been approved for the treatment of obstructive sleep apnea (OSA), a disease commonly associated with hypertension and obesity [19]. Treatment of OSA with tirzepatide may additionally mediate BP lowering [20]. In meta-analyses and post hoc analyses of clinical trials investigating the efficacy of semaglutide in people with overweight or obesity with and without diabetes, clinically meaningful BP lowering effects were observed [[21], [22], [23], [24]]. Similarly, orforglipron, an oral non-peptide GLP-1 RA, was associated with BP reduction across studies in adults with obesity or overweight with and without type 2 diabetes [[25], [26], [27], [28]]. Orforglipron is approved in the US in combination with a reduced-calorie diet and increased physical activity to reduce excess body weight and maintain weight reduction long term in adults with obesity or adults with overweight in the presence of at least one weight-related comorbid condition.

However, the above evidence for BP reduction associated with incretin-based therapies derives from secondary analyses of trials that did not enroll participants with hypertension as a primary indication [[17], [18], [19],[22], [23], [24], [25], [26], [27], [28], [29], [30]]; there are no dedicated prospective randomized trials assessing BP reduction as a primary outcome in well-characterized hypertension populations. Thus, existing evidence is limited by methodological constraints inherent to post hoc analyses, including heterogeneous and uncontrolled background antihypertensive medication use, absence of standardized BP measurement protocols, absence of ambulatory BP monitoring, end points not powered or pre-specified for BP outcomes, follow-up time not designed to characterize the trajectory and maximal extent of BP reduction over time and the lack of data on BP effect maintenance after treatment withdrawal.

ATTAIN—HYPERTENSION (ClinicalTrials.gov: NCT06948422) addresses this unmet methodological gap with a novel study design to investigate the safety and efficacy of orforglipron as a treatment to reduce BP in people with uncontrolled hypertension and overweight or obesity. The rationale, design, and baseline characteristics of this program are described within.

## 2 Methods

The ATTAIN—HYPERTENSION program is a phase 3 master protocol directing two independent, identical, multicenter, randomized, parallel-arm, double-blind, placebo-controlled studies. This program is being conducted in accordance with good clinical practice guidelines and the principles of the Declaration of Helsinki. Independent Ethics Committee or Institutional Review Board approval was received for each of the participating sites. A list of the institutional review boards is provided in the supplementary appendix (**Supplementary Table 1**). All participants provide written informed consent prior to trial participation.

### 2.1 Study participants

Participants eligible for this study are required to fulfill both hypertension-related and weight-related criteria outlined below.

Hypertension-related key inclusion criteria include uncontrolled hypertension defined as office SBP ≥ 140 mmHg and/or office DBP ≥ 90 mmHg (if DBP criteria alone is met, office SBP must be ≥130 mmHg) at screening and after the run-in period. Sites are trained to follow standardized procedures for assessment of BP, and to use validated and calibrated assessment devices evaluated by the independent scientific review process published by STRIDE-BP [31]. Further details are available in the supplementary appendix. Key exclusion criteria include office SBP ≥170 mmHg and/or office DBP ≥110 mmHg at screening or after run-in period; known secondary causes of hypertension; < 80% adherence to study-provided antihypertensive medication prior to randomization as monitored by the site investigators.

Importantly, the program prohibits enrollment of individuals initiating or adjusting medication that may impact BP within 1 month of screening. This includes medications from the following classes: angiotensin receptor blockers (ARBs) and angiotensin-converting enzyme (ACE) inhibitors, thiazide and thiazide-like diuretics, dihydropyridine calcium channel blockers. A full list of restricted and prohibited medications is included in the supplementary appendix (Supplementary Table 2).

A key overweight/obesity related eligibility criterion is a BMI ≥ 25 kg/m^2^. Key exclusion criteria include any use of GLP-1 RA or GIP/GLP-1 RA within 3 months of screening or prior or planned surgical intervention for weight reduction; and any self-reported change in body weight > 5 kg within 3 months prior to screening. A complete list of eligibility criteria is included in Supplementary Table 3.

### 2.2 Study design

The ATTAIN—HYPERTENSION program is conducted under a phase 3 master protocol comprising two independent, identical, multicenter, randomized, parallel-arm, double-blind, placebo-controlled studies evaluating the efficacy and safety of orforglipron at maximum tolerated dose (MTD) compared with placebo in adult participants with hypertension and overweight or obesity. The two studies are conducted independently, but in parallel at the same sites. Participants are randomized to one of the two studies after enrollment. Unless otherwise stated, the end points are analyzed independently for each study. As the two studies are identical in design, the following description applies to both.

Participants are randomized 2:1 to orforglipron or placebo. Study intervention used in this program are capsule formulations, which are blinded for treatment and dose. Orforglipron is approved in the US as a tablet formulation. Capsule and tablet formulations are bioequivalent, with capsule doses of 1 mg, 3 mg, 6 mg, 12 mg, 24 mg, and 36 mg corresponding to approved tablet doses of 0.8 mg, 2.5 mg, 5.5 mg, 9 mg, 14.5 mg, and 17.2 mg, respectively [32]. Participants completed gradual capsule dose escalation to reach a MTD within the 12 mg, 24 mg, and 36 mg strengths (equivalent to 9 mg, 14.5 mg, and 17.2 mg tablet strength). The 60-week study consists of 5 periods: screening, run-in, treatment, randomized withdrawal, and safety follow-up (Fig. 1).

**Fig. 1:**
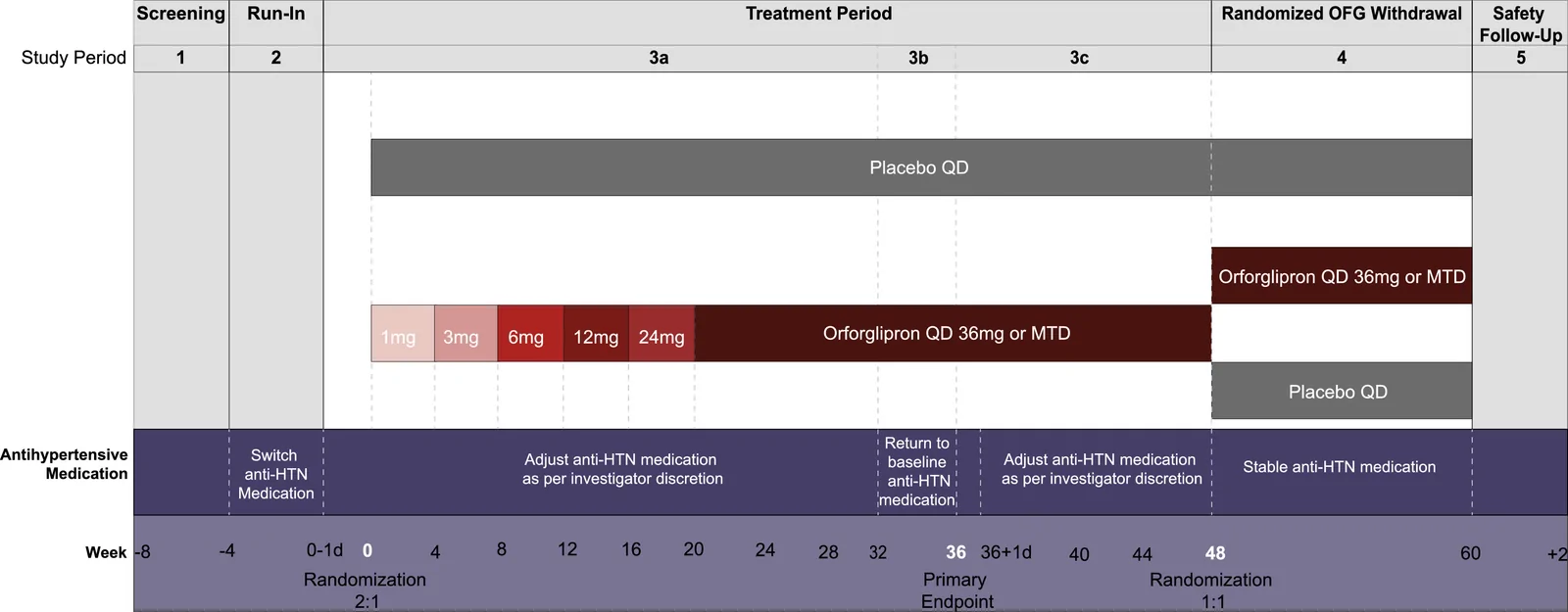
Study Design. Note: Only those participants taking antihypertensive medications at screening will complete the run-in period (Study Period 2). All other participants will proceed to randomization and treatment period after screening is complete. During Study Period 3b, participants return to their baseline antihypertensive regimen present at the time of randomization. Orforglipron is approved in the US as a tablet formulation. Capsule and tablet formulations are bioequivalent, with presented capsule doses of 1 mg, 3 mg, 6 mg, 12 mg, 24 mg, and 36 mg corresponding to approved tablet doses of 0.8 mg, 2.5 mg, 5.5 mg, 9 mg, 14.5 mg, and 17.2 mg, respectively [32]*. Abbreviations: d = day; HTN = hypertensive; MTD = maximum tolerated dose; OFG = orforglipron; QD = once daily*.

### 2.3 Screening and run-in (Study periods 1 and 2)

During the 4-week screening period, initial study eligibility is assessed. Participants who enter the study taking ARBs/ACE inhibitors, thiazides or thiazide-like diuretics, or dihydropyridine calcium channel blocker medications are classified “treated” in ATTAIN—HYPERTENSION. Treated participants complete the run-in period. Participants without preexisting antihypertensive medications within the above medication classes are considered “untreated”, do not participate in the run-in period, and progress to study period 3 (randomized treatment period) after the screening period. Antihypertensive medication of other classes are considered restricted medications that participants are instructed to remain on stable doses throughout the program.

During the 4-week run-in period, participants switch from their pre-enrollment antihypertensive medication regimen to study-provided standard antihypertensive medications of approximately equivalent potency (olmesartan, hydrochlorothiazide, or amlodipine) termed “baseline medication”. A detailed table providing examples of equivalent doses of study-provided medications for each class is included in the supplementary appendix (Supplementary Table 4).

### 2.4 Randomized treatment period (Study period 3)

Upon completion of the screening period for untreated participants, and the run-in for treated participants, if hypertension remains uncontrolled (office SBP ≥ 140 mmHg and/or office DBP ≥ 90 mmHg, and if DBP criteria alone is met, office SBP must be ≥130 mmHg) and adequate adherence (≥80% adherence to study-provided antihypertensive medication) is demonstrated, participants undergo 24-hour ambulatory blood pressure monitoring (ABPM) and are then randomized to treatment at Week 0 (baseline). The treatment period is subdivided into three distinct periods, each with specified objectives.

#### 2.4.1 Study period 3a

Participants are randomized to blinded study treatment orforglipron or placebo in a 2:1 ratio. Blinded study medication is initiated at 1 mg capsule strength (equivalent to 0.8 mg tablet strength) and escalated every 4 weeks until the MTD dose is reached. Participants are allowed 28 weeks to complete their dose escalation while optimizing tolerability. Adjustment to study-provided antihypertensive medications and doses is permitted at the investigator’s discretion during Study Period 3a.

#### 2.4.2 Study period 3b

At Week 32, participants return to the baseline antihypertensive regimen doses that were taken at the time of randomization to treatment (Week 0/ baseline) for 4 weeks. This four-week interval concludes with the primary end point assessment evaluating the change in office SBP from baseline to Week 36. Additionally a repeat 24-hour ABPM is undertaken. The protocol allows for this period to be shortened and concluded early, in the event of any safety concerns related to the adjustment of antihypertensive medications. In all cases, however, the office BP and 24-hr ABPM are completed prior to further changes in antihypertensive medication.

#### 2.4.3 Study period 3c

Study Period 3c includes an additional 12-week period during which participants accrue further exposure to blinded study treatment at MTD. During this study period, the baseline antihypertensive medication present at the time of randomization to treatment may be adjusted at the discretion of the investigator, while study treatment continues within the MTD dose range.

### 2.5 Randomized withdrawal (Study period 4)

Following completion of the 48-week treatment period, all participants remaining in the study on treatment enter a 12-week double-blind, randomized withdrawal period. Participants initially randomized to orforglipron are re-randomized 1:1 to continue MTD orforglipron or switch to placebo. Participants initially randomized to placebo will undergo a sham re-randomization and continue placebo for 12 weeks. Adjustments to study-provided antihypertensive medication are not permitted during this period; however, as in Study Period 3b, Study Period 4 may be shortened for safety issues requiring rescue or adjustment of antihypertensive medication. Blinded study medication is discontinued at the conclusion of Study Period 4.

### 2.6 Safety follow-up

All participants complete a safety follow-up visit 2 weeks after discontinuing study treatment. Participants may remain on study-provided antihypertensive medication through the safety follow-up visit. This visit marks the end of all study-related procedures and treatment.

### 2.7 Treatment

#### 2.7.1 Blinded study medication

All study participants are randomized to receive blinded, orally administered study intervention. Participants will escalate once-daily dose every 4 weeks until MTD is reached. Participants who are unable to reach a dose within the MTD range by Week 28 are discontinued from study treatment but may remain in the study. The site investigators receive detailed instructions on how to modify dose and manage treatment interruption, if necessary.

#### 2.7.2 Antihypertensive medication

Antihypertensive medication may be adjusted at the investigator’s discretion throughout specified periods of study. In cases of severe hypertension (defined as SBP >170 mmHg or DBP >110 mmHg), or for other BP values deemed clinically unacceptable by the investigator, rescue medication with dose increases of an existing, or addition of new, study-provided antihypertensive medication is permitted.

#### 2.7.3 Restricted and prohibited medication

Guidance regarding the use of medications that may either impact BP or interact with blinded study medication is provided to the investigators. A number of medications are permitted if they were started more than one month prior to screening and remain at that stable dose without adjustment throughout study participation. This includes beta blockers, mineralocorticoid receptor antagonists, non-dihydropyridine calcium channel blockers, alpha-adrenergic receptor blockers, or sodium-glucose co-transporter-2 inhibitors. Other permitted medication includes chronic (>14 days) systemic glucocorticoid therapy (except topical, intraocular, intranasal, intraarticular, interphalangeal, or inhaled preparations), sympathomimetic agents, loop diuretics, and organic nitrate preparations if initiated at least 1 month of screening.

Medications that are not permitted throughout study participation include GLP-1 RA and GIP/GLP-1 RA, hydralazine, oral minoxidil, clonidine, guanfacine, CYP3A inhibitors or CYP3A inducers, P-gp substrates, or strong organic anion-transporting polypeptide (OATP) inhibitors.

#### 2.7.4 Diet and lifestyle counseling

Sites are provided with a guide to diet and lifestyle counseling tailored for adults with overweight or obesity. This includes encouraging participants to engage in regular physical activity, such as 150 min of moderate-intensity aerobic exercise or 75 min of vigorous-intensity exercise per week and additional muscle-strengthening activities on at least 2 days per week. Additional guidance specific to participants with hypertension, including dietary sodium guidance, is also provided. Individualized diet and lifestyle counseling is offered to all participants throughout study participation via investigator’s guidance.

#### 2.7.5 Study assessments

The primary efficacy end point is the change in office SBP from baseline to Week 36. Key secondary and additional end points are summarized in Table 1. Safety assessments include adverse events, clinical laboratory findings, psycho-behavioral status, and 12-lead electrocardiograms. Participants with type 2 diabetes (T2D) will have fundus photography to assess for any changes related to study treatment.

**Table 1:** Summary of end points.

End points
**Primary**
Change in office SBP from baseline to Week 36
**Key Secondary (36-week outcomes)**
Change from baseline to Week 36
● 24-hour ABPM SBP
● Percent change body weight
● Percent change in triglycerides
● Percent change in non-HDL cholesterol
**Key Secondary (48-week outcomes)**
Percent change in hsCRP concentration (mg/L) from baseline to Week 48
**Key Secondary (Week 48 to Week 60 outcomes)**
Change in office SBP from Week 48 to Week 60 in randomized withdrawal population
**Additional Secondary**
● Change in 24-hour ABPM mean daytime SBP from baseline to Week 36 ● Change in 24-hour ABPM mean nighttime SBP from baseline to Week 36 ● Change in 24-hour ABPM mean DBP from baseline to Week 36
● Percentage of participants with office BP <140/90 mmHg at Week 36 ● Percentage of participants with office BP <130/80 mmHg at Week 36
Change from baseline to Week 36 in office DBP
Change from baseline to Week 32 in antihypertensive medication intensity
Percent change in body weight from baseline to Week 60
Change from baseline to Week 36 in Waist circumference and Waist-to-height ratio
Percent change from baseline to Week 36 in fasting total cholesterol, LDL cholesterol, and apolipoprotein B
Change in hsCRP concentration (mg/L) from baseline to Week 60
Population PK and PD parameters
Summary of safety data, including number and incidence of SAEs, TEAEs, and discontinuations due to AEs

Abbreviations: ABPM=Ambulatory blood pressure monitoring; DBP=Diastolic blood pressure; HDL=High-density lipoprotein; hsCRP=high‑sensitivity C‑reactive protein; LDL=Low-density lipoprotein; PD=Pharmacodynamic; PK=Pharmacokinetics; SAE=Serious adverse event; SBP=Systolic blood pressure; TEAE=Treatment-emergent adverse event.

Additional biomarker assessments are conducted across the entire randomized cohort, including but not limited to angiotensinogen, aldosterone, plasma renin activity, NT-proBNP, and the ambulatory arterial stiffness index derived from 24-hour ABPM data. There will be additional mechanistic assessments conducted in a subset of approximately 200 participants; this is described in the Mechanism of Action Substudy section below.

### 2.8 Mechanism of action substudy

A subset of approximately 200 participants provide consent to participate in MRI and 24-hour urine collection at baseline and at the time of the primary end point. The MRI acquisition protocol will include sequences for measurement of renal sinus fat, perirenal fat, renal fat content, visceral adipose tissue volume, abdominal subcutaneous adipose tissue volume, and liver fat content. Some participants also have extensive dietary recall interviews to aid in interpretation of 24-hour urine parameters. These data are used to explore a number of potential exploratory mechanisms by which orforglipron treatment may be beneficial in hypertension, including diet changes, leading to a reduction in sodium intake, reduction of the renin-angiotensin-aldosterone system (RAAS) activity mediated by an improvement in the fatty kidney, and increase in natriuretic peptide excretion.

### 2.9 Statistical analysis

The sample size was determined by the primary end point. A difference of at least an additional 4 mmHg reduction in mean office SBP from baseline at 36 weeks for orforglipron MTD compared with placebo, with a common SD of 13 mmHg, is assumed for statistical power calculations. This was estimated based on SBP data obtained in previous clinical trials investigating GLP-1/GIP therapies [28,33]. Under this assumption, randomizing 487 participants in each study in a 2:1 ratio to orforglipron MTD (325 participants) and placebo (162 participants) provides >89% power to demonstrate superiority of orforglipron MTD over placebo in mean change from baseline in SBP at Week 36 using a 2-sample *t*-test under the treatment regimen estimand. The greater allocation of participants to the orforglipron arm is to ensure that the study is sufficiently powered for the randomized withdrawal end point. The primary objective will be analyzed using the treatment regimen estimand and the efficacy estimand, with the efficacy estimand being considered as supportive. Within each estimand, multiplicity adjusted analyses will be performed on the primary and key secondary end points to control the familywise error rate at a 2-sided significance level of 0.05 within each study.

Under treatment regimen estimand, the primary and key secondary end points will be analyzed based on the intention-to-treat principle using randomized participants and treatment regimen estimand data points set, defined as data obtained during the treatment period at or after randomization, regardless of discontinuation of study intervention, initiation of prohibited medications or rescue therapy, or study intervention interruptions. End points will be analyzed for the two trials independently, except the key secondary end point of mean change in office SBP from Week 48 to Week 60 during the randomized withdrawal period. This end point will be evaluated using those participants originally randomized to orforglipron and re-randomized to orforglipron MTD or placebo at Week 48 by pooling data across the 2 studies. Treatment groups will be compared using an ANCOVA model controlling for baseline value and stratification factors [34]. The stratification factors include the use of antihypertensive medications at screening (yes or no), gender (individual assigned female at birth [AFAB], individual assigned male at birth [AMAB]), and the geographic region or country (the US, Europe, East Asia, and rest of world) of enrollment. Comparisons between treatment groups for binary outcomes included in additional secondary end points will be analyzed using a logistic regression model controlling for baseline value and stratification factors. Missing values at the end point of interest will be imputed using multiple imputation prior to fitting the models.

## 3 Results

The ATTAIN—HYPERTENSION program randomized 538 participants in Trial 1 and 546 in Trial 2 (Total *N* = 1084) at 100 locations across 10 countries between April 2025 and January 2026. The mean (SD) age is 53.9 (12.2) and 53.6 (11.4) years, the mean body weight is 100.7 (22.6) and 103.4 (23.5) kg, and the mean systolic BP is 144.1 (10.5) and 144.3 (10.1) mmHg at baseline, in Trial 1 and Trial 2, respectively. Half of the participants are female (48% and 51.3%), most are White (72.1% and 75.1%), and 98.5% and 98.7% of participants have a known history of hypertension, in Trial 1 and Trial 2, respectively. 53.7% of randomized participants in Trial 1 and 57.0% of randomized participants in Trial 2 are stratified as treated with antihypertensive medication at screening and entered the run-in period. The key baseline demographics and clinical characteristics of enrolled participants are presented in Table 2. There are no meaningful differences in baseline characteristics between trials.

**Table 2:** Key baseline demographics and clinical characteristics of randomized participants.

Parameter	Trial 1	Trial 2	Total
	*N* = 538	*N* = 546	*N* = 1084
**Baseline demographics**			
Age (years), mean (SD)	53.9 (12.2)	53.6 (11.4)	53.7 (11.8)
Gender female, n (%)	258 (48.0%)	280 (51.3%)	538 (49.6%)
Race, n (%)†			
White	388 (72.1%)	410 (75.1%)	798 (73.6%)
Asian	99 (18.4%)	97 (17.8%)	196 (18.1%)
Black or African American	42 (7.8%)	28 (5.1%)	70 (6.5%)
Native Hawaiian or other pacific	0	1 (0.2%)	1 (0.1%)
American Indian or Alaska native	0	0	0
Multiple	4 (0.7%)	3 (0.5%)	7 (0.6%)
Hispanic or Latino ethnic group, n (%)	154 (28.6%)	157 (28.8%)	311 (28.7%)
Country, n (%)			
United States	119 (22.1%)	120 (22.0%)	239 (22.0%)
Argentina	96 (17.8%)	101 (18.5%)	197 (18.2%)
Spain	38 (7.1%)	33 (6.0%)	71 (6.5%)
China	58 (10.8%)	60 (11.0%)	118 (10.9%)
Greece	38 (7.1%)	32 (5.9%)	70 (6.5%)
Poland	39 (7.2%)	46 (8.4%)	85 (7.8%)
Czech Republic	61 (11.3%)	63 (11.5%)	124 (11.4%)
Japan	30 (5.6%)	31 (5.7%)	61 (5.6%)
India	7 (1.3%)	5 (0.9%)	12 (1.1%)
Germany	52 (9.7%)	55 (10.1%)	107 (9.9%)
Height (cm), mean (SD)	169.2 (10.0)	169.1 (10.3)	169.1(10.1)
Body Weight (kg), mean (SD)	100.7 (22.6)	103.4 (23.5)	102.1(23.1)
BMI (kg/m^2^), mean (SD)	35.1 (6.7)	36.1 (7.0)	35.6 (6.9)
BMI category			
≥25 kg/m^2^ to <30 kg/m^2^	127 (23.6%)	104 (19.0%)	231 (21.3%)
≥30 kg/m^2^ to <35 kg/m^2^	179 (33.3%)	166 (30.4%)	345 (31.8%)
≥35 kg/m^2^ to <40 kg/m^2^	125 (23.2%)	152 (27.8%)	277 (25.6%)
≥40 kg/m^2^	107 (19.9%)	124 (22.7%)	231 (21.3%)
**Blood pressure-related baseline characteristics**			
Office SBP (mmHg), Average (SD)			
Baseline	144.1 (10.5)	144.3 (10.1)	144.2 (10.3)
Office DBP (mmHg), Average (SD)			
Baseline	89.4 (9.0)	89.7 (9.4)	89.6 (9.2)
Stratified as treated*	289 (53.7%)	311 (57.0%)	600 (55.4%)
Hypertension history	530 (98.5%)	539 (98.7%)	1069 (98.6%)
**Other clinical baseline characteristics**			
HbA1c (%), mean (SD)	5.7 (0.7)	5.8 (0.8)	5.8 (0.8)
T2D at Baseline, n (%)	114 (21.2%)	102 (18.7%)	216 (19.9%)
ASCVD History, n (%)	47 (8.7%)	55 (10.1%)	102 (9.4%)
hsCRP (mg/L), median (IQR)	2.6 (1.2, 5.0)	2.8 (1.4, 5.8)	2.8 (1.2, 5.3)
eGFR (mL/min/1.73 m^2^), mean (SD)	84.1 (19.2)	83.8 (18.8)	84 (19.0)
Lipids (mg/dL), median (IQR)			
Total cholesterol	193.7 (166.3, 223.5)	195.3 (163.2, 219.3)	194.1 (165.1, 220.4)
Triglycerides	133.7 (101.9, 184.2)	137.7 (101.9, 187.8)	136.4 (101.9, 185.1)
HDL cholesterol	46.0 (40.2, 57.2)	47.2 (40.2, 57.2)	46.4 (40.2, 57.2)
LDL cholesterol	112.7 (91.5, 139.6)	109.4 (85.8, 133.8)	111.8 (88.9, 136.9)
Non-HDL cholesterol	143.1 (119.1, 173.2)	142.3 (115.2, 170.1)	143.1 (117.2, 171.3)

Data are mean ± SD, unless otherwise stated.

⁎ Those participants stratified as treated participants who entered the run-in period. Please note that the baseline data presented here were analyzed prior to 26 May 2026 and might be subject to change while the study is ongoing.

† 12 participants have not reported race. Abbreviations: ASCVD=Atherosclerotic Cardiovascular Disease; BL=Baseline; d=day; DBP=Diastolic blood pressure; eGFR=estimated glomerular filtration rate; HbA1c=Glycated hemoglobin A1C; HDL= High-density lipoprotein; hsCRP=high‑sensitivity C‑reactive protein; LDL=Low-density lipoprotein; SBP=Systolic blood pressure; SD=Standard Deviation; T2D=Type 2 Diabetes.

## 4 Discussion

The rationale, design, and baseline characteristics of ATTAIN—HYPERTENSION, a phase 3 master protocol with two independent studies evaluating orforglipron as a primary treatment for hypertension in adults with obesity or overweight are described here. Excess adiposity is a major contributor to the development and maintenance of hypertension [5], however, no dedicated trial has investigated an incretin-based therapy with the specific purpose of treating hypertension. ATTAIN—HYPERTENSION addresses this gap through a purpose-built design that distinguishes it from prior work, including a well-characterized hypertension population, standardized background antihypertensive therapy, 24-hour ABPM, pre-specified BP end points, and a randomized withdrawal period to assess the durability of effect.

### 4.1 Study population

The BP thresholds specified in the inclusion criteria align with the classification of hypertension defined in international guidelines [[8], [9], [10]] and are consistent with those used in hypertension-focused trials [35]. Importantly, BP eligibility criteria are verified at screening and directly prior to randomization, ensuring that participants entering the treatment period have persistently elevated BP rather than transiently elevated readings at a single assessment.

The BMI inclusion criterion of ≥25 kg/m² follows the definition outlined by World Health Organization (WHO) [4], and is lower than the ≥27 kg/m² commonly applied in chronic weight management studies, which additionally require at least one weight-related comorbidity [30,36,37]^.^ This design enables assessment of orforglipron's antihypertensive and mechanistic effects across a broader BMI range. As a consequence, the average BMI in ATTAIN—HYPERTENSION is 35.6 kg/m² and understandably, but not markedly, higher than those reported in recent pivotal hypertension trials that did not include a BMI threshold, including PRECISION (33.7 kg/m²) [35], ADVANCE HYPERTENSION (31.2–32.4 kg/m²) [38], or BaxHTN (31.1–31.5 kg/m²) [39]. This speaks to the high prevalence of participants with obesity or overweight among contemporary hypertension trials. Additionally, while those trials included only participants uncontrolled on at least 2 antihypertensive medications or with resistant hypertension, ATTAIN—HYPERTENSION enrolled participants into 2 strata, treated and untreated, reflecting a broader spectrum of people who could benefit from an alternative approach for BP lowering. The inclusion of an untreated study population with uncontrolled hypertension at study start will provide evidence for the usage of orforglipron as a potential first-line treatment strategy.

### 4.2 Study design elements

#### 4.2.1 Master protocol

ATTAIN—HYPERTENSION is a master protocol directing two identical, independent trials. The use of a master protocol directing two trials provides operational efficiencies and two adequate and well-controlled trials required to establish regulatory approval while harmonizing eligibility criteria, operational conduct, and end point assessment across two studies.

#### 4.2.2 Screening and run-in

Those ATTAIN—HYPERTENSION participants considered as treated for hypertension at screening complete the run-in period while taking standardized antihypertensive treatment to establish a controlled pharmacological background to assess the BP lowering effect of orforglipron. Establishing a standard baseline therapy controls for the confounding effect of varying antihypertensive medication use between treatment groups and has been used in other hypertension treatment studies [35,38]. This period also confirms that BP remains uncontrolled under study-provided antihypertensive medication prior to randomization.

#### 4.2.3 Study period 3a

The 20-week escalation period for orforglipron titration reflects requirements to improve tolerability of adverse events like gastrointestinal symptoms commonly observed with GLP-1 RAs. Additionally, this period allows orforglipron to reach the maximum antihypertensive treatment effect expected. Orforglipron was administered in capsule formulation in this program as the study was planned prior to United States FDA approval of the tablet formulation. The capsule formulations used in this program are bioequivalent to the approved tablet formulations [32]. To ensure adequate BP control during Period 3a, adjustment of antihypertensive medication is permitted. This prevents exposing participants to sustained, inadequate antihypertensive control during dose escalation.

#### 4.2.4 Study period 3b

Given that antihypertensive medication may have been adjusted during the previous period, returning participants to their baseline antihypertensive regimen present at the time of randomization to treatment in Period 3b is critical before assessing the primary end point at Week 36. This ensures the evaluation of orforglipron's antihypertensive effect on top of the same pharmacological background that characterized uncontrolled hypertension at baseline, isolating the study drug's effect from any changes in background therapy introduced during escalation. This design mirrors the approach used in the KARDIA-1 study, which investigated zilebesiran for mild to moderate hypertension [40]. Participants' safety is maintained during this period by ensuring those participants with severe hypertension or symptomatic hypotension are not returned to baseline and advance to Period 3c. As an additional safeguard, participants who do return to baseline medication present at the time of randomization to treatment may advance to Period 3c on an accelerated timeline if unacceptable BP values are detected.

#### 4.2.5 Study period 3c

Study Period 3c permits the adjustment of antihypertension treatment while participants will continue on MTD of the blinded study treatment.

### 4.3 Randomized withdrawal period

A randomized withdrawal period is a recognized feature of hypertension studies and a regulatory requirement [41] to assess the durability of the effect and the potential consequences of treatment interruption [35]. The 6-month maintenance period at MTD prior to the randomized withdrawal meets regulatory expectations for the assessment of sustained antihypertensive efficacy in new treatments for hypertension. A 12-week randomized withdrawal period was selected to account for the uncertainty regarding the pace at which BP effects may reverse after orforglipron discontinuation, which may differ from conventional antihypertensive agents. The data for both studies under the ATTAIN—HYPERTENSION program will be pooled for analysis of the withdrawal effect. This ensures sufficient statistical power to detect the withdrawal effect while maintaining independence between the two study populations.

### 4.4 Assessments

The primary end point is change from baseline in office SBP, a standard efficacy measure for regulatory approval of antihypertensive therapies and devices [41]. The measurement of 24-hour ABPM as a key secondary end point provides additional assessment of BP, providing a full 24-hour profile, including changes in nocturnal BP, which is one of the strongest independent predictors of cardiovascular mortality and morbidity [9]. ABPM also avoids variability associated with the measurement in an office setting and provides BP readouts across activities throughout the day.

Orforglipron effectively lowers body weight and is associated with improvements in markers of cardiometabolic risk factors in people with overweight and obesity [26,27,36]. Therefore, the secondary end points also include body weight and improvement in cardiometabolic risk factors, specifically reduction of inflammatory biomarkers (hsCRP) and serum lipids (triglycerides and non-HDL cholesterol). This comprehensive assessment of improvements other than BP reflects the expectation that addressing overweight or obesity as the main driver of hypertension in the study population will simultaneously lead to additional beneficial effects in cardiometabolic parameters. This patient-centered design allows for the full characterization of the cardiometabolic benefits of orforglipron in people with hypertension and overweight or obesity, expanding on conventional hypertension trials and therapies that have not taken a holistic view to cardiovascular risk reduction.

Preclinical and post hoc clinical evidence of the SURPASS and SURMOUNT programs suggest that incretins may be involved in weight-related and weight-unrelated mechanisms leading to the improvement of cardiometabolic risk factors. However, no clinical trial has been conducted to dissect the effect of GLP-1 RAs on the mechanistic pathways associating hypertension with obesity. The mechanism of action substudy aims to address this gap. The outcomes will contribute to our understanding of whether orforglipron’s antihypertensive effect is mediated by improvements in renal fat accumulation, RAAS activation, sodium intake, and natriuretic peptide excretion. This will facilitate a clearer understanding of the contributions of weight-dependent and weight-independent mechanisms in GLP-1-mediated BP reduction. Gaining a better understanding of the mechanism involved in BP reduction represents an important advancement in our understanding of hypertension in people with overweight or obesity.

## 5 Conclusion

The ATTAIN—HYPERTENSION program establishes a novel methodical approach for the specific evaluation of orforglipron as a primary treatment for hypertension in people with overweight or obesity. In addition to investigating the efficacy and safety of orforglipron for BP reduction, the program is designed to generate evidence supporting the use of orforglipron as a first-line antihypertensive treatment in untreated people, and as an add-on therapy to existing antihypertensive treatment in those with uncontrolled hypertension. The program also characterizes the effect of orforglipron on additional cardiometabolic parameters, and the mechanism through which BP is reduced.

Central Illustration.

**Figure:**
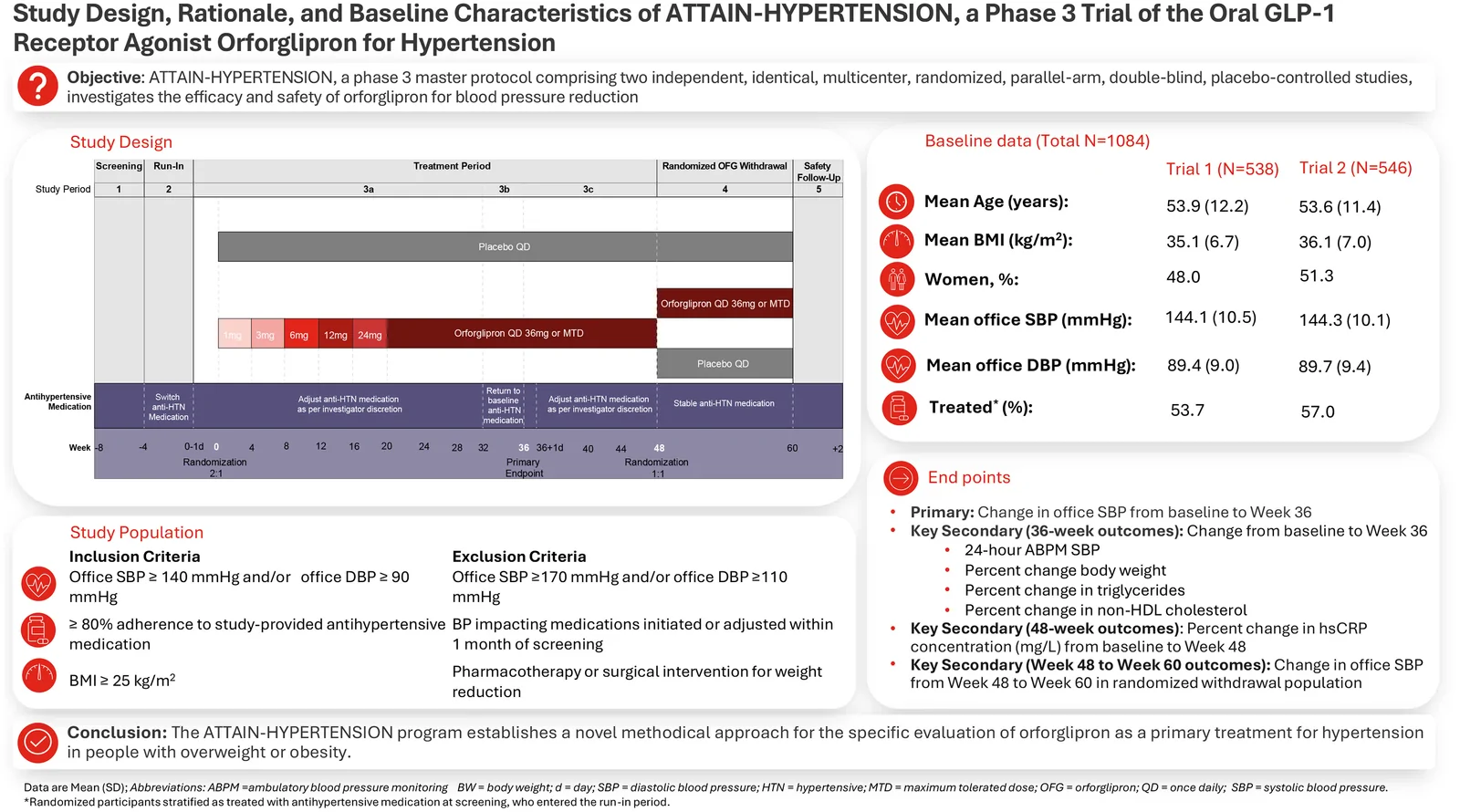

## Funding

This study was supported by 10.13039/100004312Eli Lilly and Company.

## CRediT authorship contribution statement

**Luke Laffin:** Writing – review & editing, Conceptualization. **Markus P. Schlaich:** Writing – review & editing, Conceptualization. **Michael E. Hall:** Writing – review & editing, Conceptualization. **Cathy C. Xie:** Writing – review & editing, Writing – original draft, Validation, Methodology, Formal analysis, Data curation, Conceptualization. **Girish Rao:** Writing – review & editing, Writing – original draft, Conceptualization. **Mallik Rettiganti:** Writing – review & editing, Conceptualization. **Ellen B. Dennehy:** Writing – review & editing, Writing – original draft, Visualization, Supervision, Methodology, Data curation, Conceptualization. **Bruno Linetzky:** Writing – review & editing, Writing – original draft, Visualization, Supervision, Project administration, Methodology, Investigation, Data curation, Conceptualization.

## Declaration of competing interest

The authors declare the following financial interests/personal relationships which may be considered as potential competing interests:

Luke Laffin reports administrative support, article publishing charges, statistical analysis, and writing assistance were provided by 10.13039/100004312Eli Lilly and Company. Markus Schlaich reports financial support was provided by 10.13039/100004312Eli Lilly and Company. Michael E. Hall reports financial support was provided by 10.13039/100004312Eli Lilly and Company. Luke Laffin reports a relationship with 10.13039/501100012408Recor that includes: consulting or advisory. Luke Laffin reports a relationship with 10.13039/100004374Medtronic that includes: consulting or advisory. Luke Laffin reports a relationship with Mineralys Therapeutics, Inc. that includes: funding grants. Luke Laffin reports a relationship with CRISPR Therapeutics Inc that includes: funding grants. Luke Laffin reports a relationship with 10.13039/100004336Novartis that includes: consulting or advisory. Luke Laffin reports a relationship with 10.13039/501100004191Novo Nordisk that includes: consulting or advisory. Luke Laffin reports a relationship with Ripple Medical that includes: consulting or advisory. Luke Laffin reports a relationship with AstraZeneca Pharmaceuticals LP that includes: consulting or advisory. Luke Laffin reports a relationship with Arrowhead Pharmaceuticals Inc that includes: funding grants. Luke Laffin reports a relationship with Retension that includes: funding grants. Luke Laffin reports a relationship with Kardigan Inc that includes: funding grants. Luke Laffin reports a relationship with Madrigal Pharmaceuticals Inc that includes: funding grants. Luke Laffin reports a relationship with Diamedica that includes: funding grants. Luke Laffin reports a relationship with Editas Medicine that includes: funding grants. Luke Laffin reports a relationship with Kylonova Biopharma that includes: funding grants. Markus Schlaich reports a relationship with Medtronic Inc that includes: consulting or advisory and speaking and lecture fees. Markus Schlaich reports a relationship with Abbott Laboratories Inc that includes: consulting or advisory, funding grants, and speaking and lecture fees. Michael E. Hall reports a relationship with 10.13039/100004312Eli Lilly and Company that includes: consulting or advisory. Cathy C. Xie reports a relationship with 10.13039/100004312Eli Lilly and Company that includes: employment and equity or stocks. Girish Rao reports a relationship with 10.13039/100004312Eli Lilly and Company that includes: employment and equity or stocks. Mallik Rettiganti reports a relationship with 10.13039/100004312Eli Lilly and Company that includes: employment and equity or stocks. Ellen B. Dennehy reports a relationship with 10.13039/100004312Eli Lilly and Company that includes: employment and equity or stocks. Bruno Linetzky reports a relationship with 10.13039/100004312Eli Lilly and Company that includes: employment and equity or stocks. If there are other authors, they declare that they have no known competing financial interests or personal relationships that could have appeared to influence the work reported in this paper.

## Data Availability

Lilly provides access to all individual participant data collected during the trial, after anonymization, with the exception of pharmacokinetic or genetic data. Data are available to request 6 months after the indication studied has been approved in the US and EU and after primary publication acceptance, whichever is later. No expiration date for data requests is currently set once data are made available. Access is provided after a proposal has been approved by an independent review committee identified for this purpose and after receipt of a signed data sharing agreement. Data and documents, including the study protocol, statistical analysis plan, clinical study report, and blank or annotated case report forms, will be provided in a secure data sharing environment. For details on submitting a request, see the instructions provided at http://www.vivli.org.
