# Supplementary material for: Study design, rationale, and baseline characteristics of ATTAIN-HYPERTENSION, a phase 3 trial of the oral GLP-1 receptor agonist orforglipron for hypertension
Source: Am J Prev Cardiol. 2026 Aug 6;30:101783. doi: 10.1016/j.ajpc.2026.101783 (PMC13633904; doi:10.1016/j.ajpc.2026.101783)
Supplement: Supplementary file 1 [file mmc1.pdf]

SUPPLEMENTARY APPENDIX

Supplement to: Laffin et al. Study Design, Rationale, and Baseline Characteristics of ATTAIN-HYPERTENSION, a Phase 3 Trial of the oral GLP-1 Receptor Agonist Orforglipron for Hypertension

Table of Contents

Supplementary Table 1. List of institutional review boards 3
Supplementary Table 2. List of restricted and prohibited medications 5
Supplementary Table 3. Inclusion and exclusion criteria 7
Guidelines for Blood Pressure Measurement 11
Examples of proposed equivalent study-provided antihypertensive medication for baseline antihypertensive medication 12

Supplementary Table 1. List of institutional review boards

Country	IRB Name
Argentina	Iniciativa y Reflexión bioética Rosario
	Comite de Etica San Isidro
	Comite de etica en investigacion de la Fundación Dr. J. R. Villavicencio
	Comité de Etica en Investigación Clínica - CEIC
	Comité de Etica e Investigación del Centro de Investigaciones Clínicas del Litoral (CICLI)
	Comite de Etica de Instituto de Investigaciones Clinicas Cordoba
China	Wuxi People's Hospital Clinical Trials Ethics Committee
	The First Affiliated Hospital of Henan University of Science and Technology IRB
	Shaanxi People's Hospital Medical Ethics Committee
	Lishui Central Hospital Ethics Committee
	Jinan Central Hospital Medical Ethics Committee
	Ethics Committee of the Second Affiliated Hospital of Chongqing Medical University
	Ethics Committee of the First People's Hospital of Changde City
	Ethics Committee of Siping Central People's Hospital
	Ethics Committee of China-Japan Union Hospital of Jilin University
	Ethics Committee of Beijing Pinggu Hospital
	EC of Sichuan Provincial People's Hospital
	Clinical Trial Ethics Committee of Ruijin Hospital Affiliated to Shanghai Jiao Tong University
Czech Republic	Eticka komise SUKL
Germany	Ethik-Kommission der Landesärztekammer Rheinland-Pfalz
Greece	National Ethics Committee
	G. Gennimatas Hospital Scientific Committee
	ERB of University Hospital of Ioannina
	Dioikitiko Simvoulio AHEPA
India	Lifecare Hospital Institutional Review Board
	INSTITUTIONAL ETHICS COMMITTEE, Bhubaneswar
	Institutional Ethics Committee
	Ashirwad Ethics Committee
Japan	Tokyo-Eki Center-building Clinic Institutional Review Board

	Review Board of Human Rights and Ethics for Clinical Studies<br>Nihonbashi Sakura Clinic IRB committee<br>Medical Corporation Yuga Higashirinkan Kaneshiro Diabetes Clinic IRB
Poland	Naczelna Komisja Bioetyczna<br>Komisja Bioetyczna przy Okręgowej Izbie Lekarskiej w Tarnowie
Spain	Comité Ético de Investigación Clínica León<br>CEIm Provincial de Córdoba
United States	St. Agnes Healthcare<br>LifeBridge Health IRB<br>Advarra

Supplementary Table 2. List of restricted and prohibited medications

**Restricted Concomitant Medications***
Drugs that may be affected by an increase in gastric pH should be separated from orforglipron administration by at least 2 to 4 hours (for example, Antihepatitis C, Antiretroviral, Tyrosine kinase inhibitors, and other narrow therapeutic index substrates with potential pH-dependent solubility or stability)
Beta blockers
Mineralocorticoid receptor agonist or other diuretics (beyond thiazide and thiazide-like diuretics)
Non-dihydropyridine calcium channel blockers (for example, verapamil and diltiazem)
Alpha-adrenergic receptor blockers (alfuzosin, doxazosin, and tamsulosin)
Estrogen-replacement or testosterone-replacement
Estrogen- or progesterone-containing oral contraceptives
Sodium-glucose co-transporter 2 inhibitors
Erythropoiesis-stimulating agents
Psychoactive drugs associated with potential weight gain (Imipramine, Amitriptyline, Mirtazapine, Paroxetine, Phenelzine, Chlorpromazine, Thioridazine, Clozapine, Olanzapine, Quetiapine, Valproic acid and its derivatives, and Lithium)
Chronic use of systemic NSAIDs, other than low dose aspirin (81-325 mg)†
Theophylline
Daily use of phosphodiesterase type 5 inhibitors (intermittent dosing not permitted), and intramuscular steroids
**Prohibited Concomitant Medications**
Change of baseline antihypertensive medication to medication(s) other than study-provided options from the 3 defined classes (ARB/ACE inhibitors, thiazides and thiazide-like diuretics, and dihydropyridine calcium channel blockers)
GLP-1 receptor agonists, GIP/GLP-1 receptor agonists, GLP-1/glucagon receptor agonists, or GIP/GLP-1/glucagon receptor agonists (Dulaglutide, Liraglutide, Exenatide, Tirzepatide, Semaglutide
Strong CYP3A inducers or inhibitors, drugs that are sensitive P-gp substrates, or strong inhibitors of OATPs (for examples Strong CYP3A4 inhibitors or inducers (Antihepatitis C, Antiretroviral, Antifungal, Antibiotic and others), Strong OATP inhibitors (Antihepatitis C, Antiretroviral, Other) and P-gp substrates with narrow therapeutic index (Antirejection and others)‡
Sympathomimetics (any formulation), including but not limited to<br>• Nasal decongestants containing phenylephrine hydrochloride, pseudoephedrine naphazoline hydrochloride§<br>• Stimulants: amphetamines and cocaine
Systemic glucocorticoid therapy (exceptions: Topical, intraocular, intranasal, intraarticular, interphalangeal, or inhaled preparations)

Furosemide ≥40 mg/day or equivalent doses of loop diuretics[||]
Organic nitrate preparations, such as nitroglycerin, isosorbide mononitrate, isosorbide dinitrate, and pentaerythritol
Herbal supplements with potential impact on blood pressure or weight, such as ephedra, ma huang, Yohimbine, St. John's wort
Licorice
Hydralazine, oral minoxidil, clonidine, and guanfacine
Insulin[¶]
Medications intended to promote weight loss, including prescribed, over-the-counter, or alternative remedies (for example, Liraglutide, Orlistat, Sibutramine, Phenylpropanolamine, Mazindol, Phentermine, Lorcaserin, Phentermine/topiramate, Naltrexone/bupropion, Tirzepatide, Semaglutide, Ingested material that transiently occupies space in the stomach, for example, Plenity®, Over-the-counter medications, for example, allī®)

*Medications are permitted if they were initiated >1 month prior to screening and dose was stable for ≥1 month prior to screening and remained stable throughout the study

†Chronic defined as > 3 consecutive or nonconsecutive days of treatment per week

‡In some circumstances strong CYP3A4 inhibitors may be used for a short duration of time (for example, for the treatment of an infection), if necessary. If this need arises, participants should undergo a temporary study intervention interruption and not reinitiate study intervention until at least 14 days after the end date of the strong CYP3A4 inhibitor.

§Short-term, intermittent use of nasal decongestants permitted, but prohibited 5 days prior to any study visit.

||Short-term use for up to 14 days is allowed for certain clinical situations, for example, for acute pulmonary edema or hospitalization for heart failure.

¶Short-term insulin use for up to 14 days is allowed for certain clinical situations, for example, elective surgery or during hospitalization.

Abbreviations: ACE=Angiotensin-converting enzyme (ACE); ARB=Angiotensin receptor blockers; CYP3A=Cytochrome P450, family 3, subfamily A; GIP=glucose-dependent insulinotropic polypeptide; GLP-1=Glucagon-like peptide-1; NSAID=Nonsteroidal anti-inflammatory drugs; OATP=Organic anion-transporting polypeptide; P-gp= P-glycoprotein.

Supplementary Table 3. Inclusion and exclusion criteria

**Key inclusion criteria**	
	Adult male or female ≥ 18 years
	SBP ≥ 140 mmHg and/or DBP ≥ 90 mmHg (if DBP criteria alone is met, SBP must be ≥130 mmHg) at Screening and Prior to Randomization
	Untreated for hypertension, or on stable antihypertensive medications ≥ 30 days prior to screening from any or all of the following classes*: • ARB/ACE inhibitors • thiazides, and thiazide-like diuretics • calcium channel blockers
	BMI ≥ 25 kg/m^2
	Individuals of childbearing potential are willing to follow recommendations for contraceptive/barrier use[†]
	Provide informed, written consent
**Key exclusion criteria**	
Hypertension	
	SBP ≥170 mmHg and/or DBP ≥110 mmHg
	Presence of secondary causes of hypertension (for example, primary hyperaldosteronism, renal artery stenosis, uncontrolled or untreated hyperthyroidism, uncontrolled or untreated hypothyroidism, hyperparathyroidism, pheochromocytoma, Cushing's syndrome, or aortic coarctation)
	Obstructive sleep apnea treated with positive airway pressure (PAP) therapy.
	Orthostatic hypotension[‡]
	Prior (≤1 year) or planned renal denervation
Cardiovascular	
	Heart failure with reduced ejection fraction (HFrEF)
	Atrial fibrillation (Exception: Paroxysmal atrial fibrillation)
	Prior conditions: • hospitalization for hypertension or for congestive heart failure, or • acute coronary syndrome or acute myocardial infarction, or • cerebrovascular accident (stroke)
	Prior (within 3 months prior to screening) or planned coronary revascularization
Diabetes	
	Type 1 Diabetes
	HbA1C >9% as determined and reported by central lab measurement
	Prior or planned treatment for diabetic retinopathy and/or macular edema[§]
	Treatment with insulin within 90 days prior to screening

Other medical
History of clinically significant gastric emptying abnormality (e.g., severe gastroparesis, gastric outlet obstruction) or chronic use of medication that directly affects gastrointestinal motility
Acute or chronic hepatitis or other liver disease (excluding nonalcoholic fatty liver disease) or ALT or AST level > 3× ULN for the reference range, ALP level ≥1.5× ULN, or total bilirubin ≥ 1.5× ULN, except for Gilbert's syndrome
Renal impairment eGFR < 30 mL/min/1.73 m^2
History of significant active or unstable major depressive disorder or other severe psychiatric disorder (e.g., schizophrenia, bipolar disorder, other serious mood or anxiety disorder) within the last 2 years or in the investigator’s opinion, have any significant mental health disorder that may put the individual at higher risk of study participation[‖]
History of active or untreated malignancy or remission from clinically significant malignancy (other than basal or squamous cell skin cancer, in situ carcinomas of the cervix, or in situ prostate cancer) for < 5 years
Calcitonin level of ≥35 ng/L (≥35 pg/mL), as determined by the central laboratory at screening
Uncontrolled endocrine abnormality in the opinion of the investigator (e.g., thyrotoxicosis, adrenal crises)
Chronic or acute pancreatitis any time prior to screening
Evidence of a significant, active autoimmune abnormality (for example, lupus or rheumatoid arthritis) that is likely to require, in the opinion of the investigator, such treatment with systemic corticosteroids during the study
Prior/concomitant therapy
Use of GLP-1 receptor agonists within 90 days of screening
Receiving or have received within 1 months prior to screening chronic systemic glucocorticoid therapy (excluding topical, intraocular, intranasal, intra-articular, interphalangeal, or inhaled preparations)
Current treatment with hydralazine, oral minoxidil, clonidine, or guanfacine
Current treatment with strong CYP3A inhibitors or CYP3A inducers, P-gp substrates, or strong OATP inhibitors
Medications that may impact BP, and/or body weight, within 1 month of screening[¶]<br>• Beta blockers<br>• Mineralocorticoid receptor antagonists or other diuretics (beyond thiazide and thiazide-like diuretics)<br>• Non-dihydropyridine calcium channel blockers (for example, verapamil and diltiazem)

- Alpha-adrenergic receptor blockers (for example, alfuzosin, doxazosin and tamsulosin)
- Estrogen-replacement or testosterone-replacement
- Estrogen- or progesterone-containing oral contraceptives
- Sodium-glucose co-transporter-2 inhibitors
- Erythropoiesis-stimulating agents
- Psychoactive drugs associated with potential weight gain
- Chronic use of systemic NSAIDs, other than low dose aspirin (81-325 mg), where chronic use is defined as > 3 consecutive or nonconsecutive days of treatment per week
- Theophylline
- Daily phosphodiesterase Type 5 inhibitors (intermittent dosing not permitted)
- Intramuscular steroids

---

*Participants who are on stable doses of beta blockers (such as metoprolol or atenolol) or mineralocorticoid receptor antagonists (such as spironolactone), and who intend to remain on these medications at existing doses, can be enrolled in the study.
†Contraceptive use by participants should be consistent with local regulations regarding the methods of contraception for those participating in clinical studies.
‡Decrease in SBP ≥ 20 mmHg or DBP ≥ 10 mmHg from sitting to standing position.
§All participants with T2D will have a baseline fundus photography assessment prior to randomization. In the presence of any degree of diabetic retinopathy and/or macular edema, further ophthalmologic assessment will follow local standards of care to confirm eligibility.
||Participants with major depressive disorder or generalized anxiety disorder whose disease state is considered stable for the past 2 years and expected to remain stable throughout the course of the study, in the opinion of the investigator, may be considered for inclusion if they are not on excluded medications.
¶Participants are eligible for the study if the listed medications were started more than 1 month prior to screening, dose has been stable for at least 1 month and is expected to remain stable throughout the study duration.
Abbreviations: ACE=Angiotensin-converting enzyme (ACE); ALP=Alkaline phosphatase; ALT=Alanine aminotransferase; ARB=Angiotensin receptor blockers; AST=Aspartate aminotransferase; BMI=Body mass index; BP=Blood pressure; CYP3A=Cytochrome P450, family 3, subfamily A; DBP=Diastolic blood pressure; eGFR=estimated glomerular filtration rate; GLP-1=Glucagon-like peptide-1; HbA1c=Glycated hemoglobin A1C; NSAID=Nonsteroidal anti-inflammatory drugs; OATP=Organic anion-transporting

polypeptide; P-gp= P-glycoprotein; SBP=Systolic blood pressure; ULN-Upper limit of normal.

Guidelines for Blood Pressure Measurement

Obtaining consistent and accurate BP measurements is critical for this study and must follow standardized procedures and use validated and calibrated assessment devices evaluated by the independent scientific review process published by STRIDE-BP(31). To follow the standardized procedure, office BP should be measured after participants have been sitting at least 5 minutes, using automated blood pressure measurement and prior to obtaining an ECG tracing and collecting blood samples. Office BP should be measured in both arms at the screening visit to detect interarm differences, and the arm with the highest BP should be used throughout the study if no indication of measuring the other arm is present. The measurement should be scheduled for a similar time of day as previous visits, and participants will be advised to take antihypertensive medication and blinded study treatment as usual.

To obtain accurate BP data, the measurement should be conducted three times on the same arm with 1-minute intervals, and the average of the three readings will determine office blood pressure. Each of the three measurements of sitting office BP needs to be recorded and documented on the case report form.

Additionally, BP must be measured in a standing position at the screening visit and, if indicated during the following visits, to monitor orthostatic hypotension. The standing BP should be averaged from two measurements taken 1 and 3 minutes after standing up, immediately after completing the sitting BP measurement.

Examples of proposed equivalent study-provided antihypertensive medication for baseline antihypertensive medication

Supplementary Table 4. Proposed equivalence of study-provided antihypertensive medication for each class of baseline antihypertensive drug

**Proposed Equivalence for ARB/ACE**				
ARB (mg)	Study-Provided Equivalent Olmesartan 20 mg		Study-Provided Equivalent Olmesartan 40 mg	
Olmesartan	20		40	
Azilsartan	40		80	
Candesartan	8	16	24	32
Irbesartan	150		300	
Losartan	50		100	
Telmisartan	20	40	60	80
Valsartan	80	160	240	320
ACE (mg)				
Benazepril	10	20	30	40
Cilazapril	2.5	5	7.5	10
Captopril	37.5	75	112.5	150
Enalapril	5	10	20	40
Fosinopril	10	20	30	40
Lisinopril	10	20	30	40
Perindopril	4	8	12	16
Quinapril	5	10	20	40
Ramipril	2.5	5	10	20
Trandolapril	1	2	3	4

**Proposed Equivalence for Thiazides and Thiazide-Like Diuretics**			
Thiazides and Thiazides Like Diuretics (mg)	Study-Provided Equivalent = Hydrochlorothiazide 12.5 mg	Study-Provided Equivalent = Hydrochlorothiazide 25 mg	Study-Provided Equivalent = Hydrochlorothiazide 50 mg
Hydrochlorothiazide	12.5	25	50
Chlortalidone		12.5	25
Indapamide		1.25	2.5
Metolazone		2.5	5

**Proposed Equivalence for Dihydropyridine Calcium Channel Blockers**			
Dihydropyridine Calcium Channel Blockers	Study-Provided Equivalent = Amlodipine 2.5 mg	Study-Provided Equivalent = Amlodipine 5 mg	Study-Provided Equivalent = Amlodipine 10 mg
Amlodipine	2.5	5	10
Felodipine	2.5	5	10

Nifedipine	30	30	60	90
